# Approaching the mapping limit with closed-loop mapping strategy for deploying neural networks on neuromorphic hardware

**DOI:** 10.3389/fnins.2023.1168864

**Published:** 2023-05-18

**Authors:** Song Wang, Qiushuang Yu, Tiantian Xie, Cheng Ma, Jing Pei

**Affiliations:** Department of Precision Instrument, Center for Brain-Inspired Computing Research (CBICR), Tsinghua University, Beijing, China

**Keywords:** neuromorphic chip, logical mapping, physical mapping, mapping limit, closed-loop mapping

## Abstract

The decentralized manycore architecture is broadly adopted by neuromorphic chips for its high computing parallelism and memory locality. However, the fragmented memories and decentralized execution make it hard to deploy neural network models onto neuromorphic hardware with high resource utilization and processing efficiency. There are usually two stages during the model deployment: one is the logical mapping that partitions parameters and computations into small slices and allocate each slice into a single core with limited resources; the other is the physical mapping that places each logical core to a physical location in the chip. In this work, we propose the mapping limit concept for the first time that points out the resource saving upper limit in logical and physical mapping. Furthermore, we propose a closed-loop mapping strategy with an asynchronous 4D model partition for logical mapping and a Hamilton loop algorithm (HLA) for physical mapping. We implement the mapping methods on our state-of-the-art neuromorphic chip, TianjicX. Extensive experiments demonstrate the superior performance of our mapping methods, which can not only outperform existing methods but also approach the mapping limit. We believe the mapping limit concept and the closed-loop mapping strategy can help build a general and efficient mapping framework for neuromorphic hardware.

## 1. Introduction

Deep neural networks (DNNs) have made a series of breakthroughs in many fields. With the exponential growth (Vaswani et al., 2017; Gholami et al., 2021) of parameters and computations of DNN models, the memory and computational costs are unaffordable for conventional (Von Neumann, 1993) architectures. To overcome the memory wall problem, the decentralized manycore architecture emerges in recent years for performing neural network workloads, which presents massive processing parallelism, memory locality, and multi-chip scalability (Painkras et al., 2013; Akopyan et al., 2015; Han et al., 2016; Jouppi et al., 2017; Parashar et al., 2017; Shin et al., 2017; Davies et al., 2018; Chen et al., 2019; Pei et al., 2019; Shao et al., 2019; Deng et al., 2020; Zimmer et al., 2020). Each functional core contains independent computation and memory resources with close distance, and cores communicate through a flexible routing fabric (Wu et al., 2020). Due to the limited hardware resources in each core, a large neural network model has to be partitioned and mapped onto cores during deployment. The mapping process experiences two stages: logical mapping and physical mapping.

In the logical mapping stage, the requirements for computation and memory resources are important consideration factors for allocating cores. The parameters and the associated computations are divided into small slices through tensor dimension partition and each slice is allocated into a single core with limited hardware resources (Shao et al., 2019; Deng et al., 2020; Wu et al., 2020). For a convolutional layer, most previous work adopts the 2D partition to split the input channel (*C*_*in*_) and the output channel (*C*_*out*_) dimensions. However, partitioning the input channel dimension would generate partial sums (*psum*), which might degrade the model accuracy. To avoid the accuracy loss, the bit-width of *psum* has to be enlarged, which unfortunately results in longer communication latency and larger memory overhead.

The logical mapping only partitions a neural network and allocates the partitioned sub-networks to cores logically. This stage does not care the physical locations of cores on real hardware. The physical mapping places each logical core to a physical location in the chip, which greatly affects the communication latency and might cause the deadlock problem (Wu et al., 2020). The core placement optimization for minimized latency is actually an NP-hard problem (Myung et al., 2021) and the search space grows rapidly as the number of cores increases. Existing algorithms for physical mapping on a 2D mesh topology are usually heuristic.

In this work, we find that there exists a limit in mapping a neural network model onto the decentralized multi-core architecture widely used by neuromorphic hardware. To approach this limit for fully utilizing hardware resources, we propose the closed-loop mapping strategy. Specifically, in logical mapping, we propose an asynchronous 4D partition between input activations (IA) and weights (W) from four dimensions for reducing execution latency; in physical mapping, we propose a Hamilton Loop Algorithm (HLA) for deadlock-free core placement with reduced communication latency. With our optimization, the running speed and computing efficiency can be improved by 7.6 and 8.8×, respectively via the integration of the logical mapping and the physical mapping, compared with the synchronous partition. Moreover, the running speed and computing efficiency can approach the performance limit of hardware, which is validated on the TianjicX chip (Deng et al., 2018).

## 2. Preliminaries and related works

### 2.1. Graph representation

**Figure 1:**
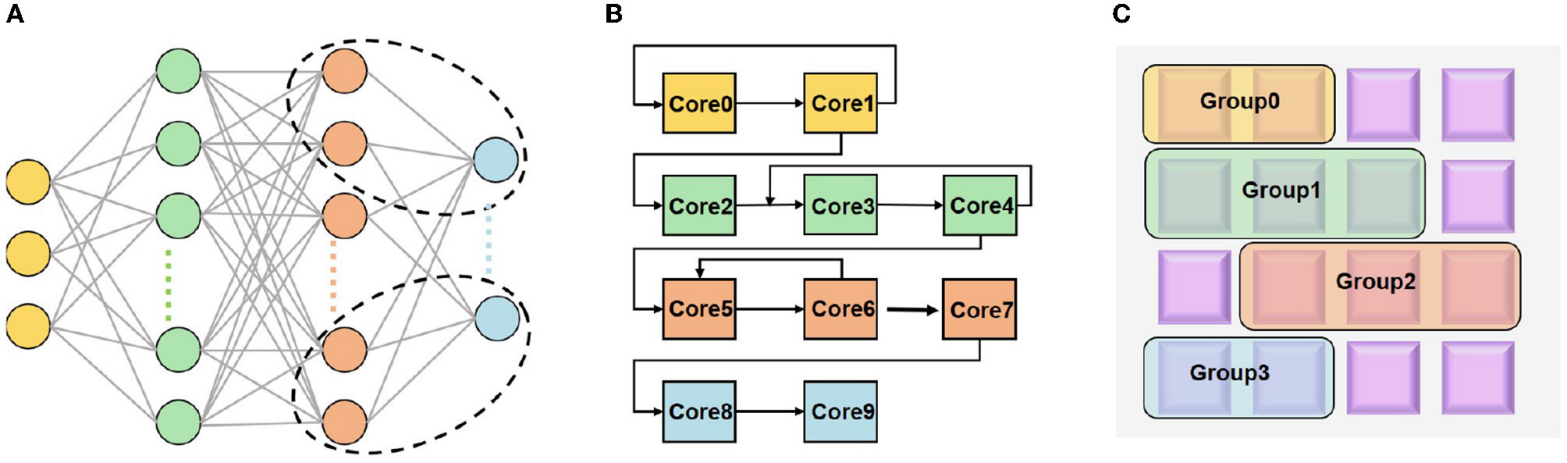
Illustration of mapping **(A)** a neural network onto neuromorphic hardware with two stages: **(B)** logical mapping; **(C)** physical mapping.

As aforementioned, mapping a neural network model onto a decentralized multi-core architecture has two stages: the logical mapping and the physical mapping, as illustrated in Figure 1.

The logical cores can be represented by a graph *G*(*V, E*), thus the physical mapping can be viewed to place *G*(*V, E*) on a circuit graph *T*(*U, S*). *V* and *U* denote the sets of nodes, i.e., logical cores and physical cores, respectively; *E* and *S* denote the sets of edges, i.e., connections between logical cores and physical cores, respectively. Specifically, the physical mapping can be described as follows:


(1) $$V\to U,\;s.t.=\{\begin{array}{l} |V\text{|}\leq \text{|}U\text{|},\\ \forall v_{i}\in V,map(v_{i})\in U,\\ \forall v_{i}\neq v_{j}\in V,map(v_{i})\neq map(v_{j}). \end{array}$$

where *v*_*i*_ and *v*_*j*_ denote the *i*-th and *j*-th nodes (i.e., core) in *V*, respectively; |*V*| and |*U*| represent the numbers of logical and physical cores, respectively. Above constraints imply one-to-one mapping from logical cores to physical cores. Furthermore, we denote the weighted edges (#packets) between *v*_*i*_ and *v*_*j*_ as *c*_*ij*_ and denote the Manhattan distance between *map*(*v*_*i*_) and *map*(*v*_*j*_) as *M*_*ij*_, i.e., *M*_*ij*_ = |*x*_*i*_ − *x*_*j*_| + |*y*_*i*_ − *y*_*j*_| where (*x*_*i*_, *y*_*i*_) and (*x*_*j*_, *y*_*j*_) are the coordinates of the two physical cores on the 2D physical plane. Let's define *E*|*h*| as the energy of transmitting a routing packet through a single hop distance, and define *L*_*ij*_ as the communication latency with a routing packet between *map*(*v*_*i*_) and *map*(*v*_*j*_), respectively. With the above definitions, the total communication cost *C*_*cost*_ (Myung et al., 2021), the average communication latency *L* (Amin et al., 2020), and the average power consumption *P* (Pei et al., 2019; Ma et al., 2020) can be calculated by


(2) $$\begin{array}{l} C_{cost}=\sum_{\forall v_{i},v_{j}\in V}c_{ij}\times M_{ij}, \end{array}$$

(3) $$\begin{array}{l} L=avg\left(\frac{1}{N_{i}}\sum_{j}c_{ij}\times L_{ij}\right), \end{array}$$

(4) $$P=\frac{C_{cost}\times E\left|\begin{array}{c} h \end{array}\right|}{T}.$$

where the *N*_*i*_ is the number of routing packets received by the physical core *map*(*v*_*i*_). The working period *T* can be approximately viewed as a fixed variable.

### 2.2. Logical mapping

**Figure 2:**
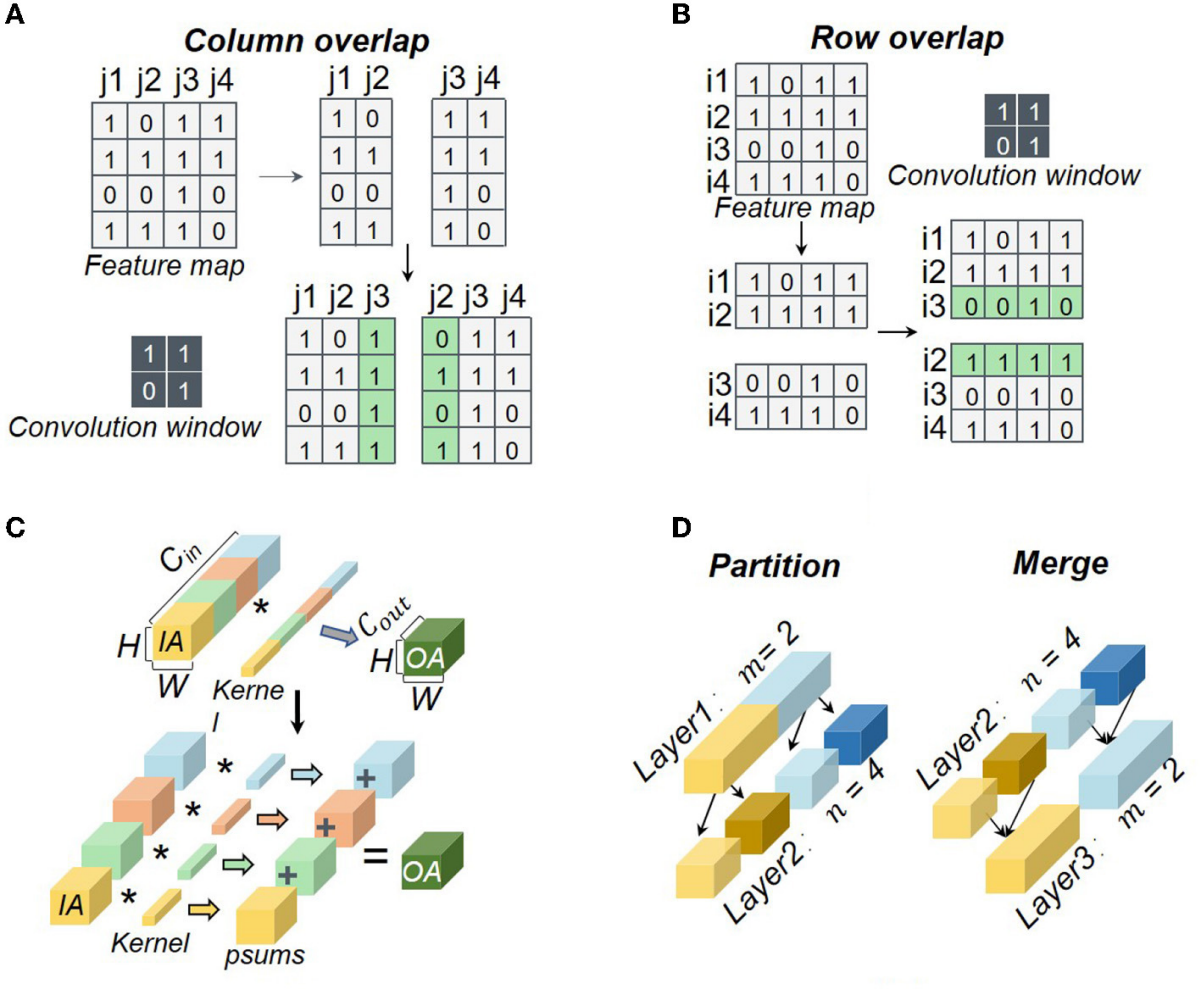
Typical challenges in neural network partition: **(A)** column-wise overlap; **(B)** row-wise overlap; **(C)** *psum* accumulation; **(D)** data reshaping. The symbol ^*^ means convolution.

At present, most researchers adopt 2D partition in logical mapping by splitting both input and output channel dimensions. The partition of the input channel dimension would compromise accuracy due to the accumulation of *psum*s, while the partition of the output channel dimension would cause the requirement for data reshaping in the next layer. Besides the 2D partition, some works such as Simba (Shao et al., 2019; Zimmer et al., 2020) and Tianjic (Pei et al., 2019; Deng et al., 2020) can support 4D partition further on the feature map width and height. However, current mapping strategies face some challenges as shown in Figure 2, including the data overlap between the feature map partition, the *psum* accumulation in the input channel partition, and the data reshaping in the output channel partition.

The additional storage overheads in a core can be generated by the row-wise overlap *S*_*row*_*add*_, the column-wise overlap *S*_*col*_*add*_, the *psum* *S*_*p*_*add*_, and the reshaped data *S*_*re*_*add*_, which also result in additional computation latency. The additional storage and computation latency can be obtained by


(5) $$\begin{array}{l} S_{add}=S_{row\text{\_}add}+S_{col\text{\_}add}+S_{p\text{\_}add}+S_{re\text{\_}add}, \end{array}$$

(6) $$\begin{array}{l} t_{add\_1}=f_{1}(S_{row\_add})+f_{2}(S_{col\_add})\\ \;+f_{3}(S_{p\_add})+f_{4}(S_{re\_add}). \end{array}$$

where *S*_*add*_ denotes the additional storage overhead in a core. *f*_*i*_(·) represents the function for processing the additional data. Note that we have *f*_*i*_(0) = 0. *t*_*add*_1_ is the additional computation latency of a core neglecting the additional latency caused by the physical mapping. Because the partition depth of input channels is equal to that of each weight filter, all of these partition methods are viewed as the synchronous partition in this work.

### 2.3. Physical mapping

**Figure 3:**
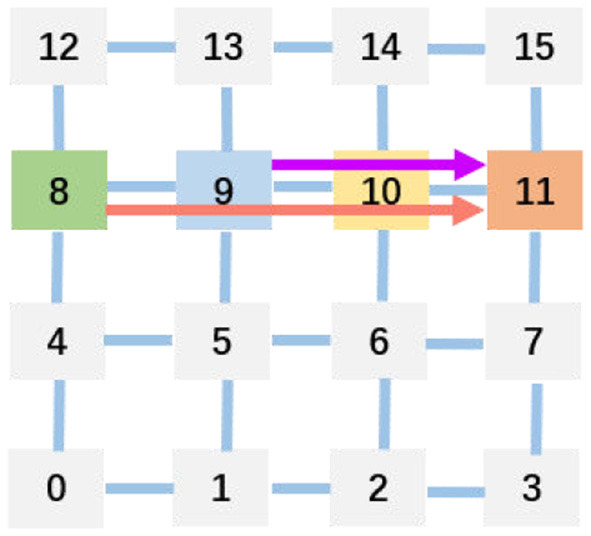
Example for helping understand communication latency caused by physical mapping.

The optimal physical mapping is acknowledged to be an NP-hard problem (Myung et al., 2021). The 2D mesh topology is widely adopted by neuromorphic hardware owing to its high throughput and scalability (Painkras et al., 2013; Akopyan et al., 2015; Davies et al., 2018; Pei et al., 2019; Shao et al., 2019; Deng et al., 2020; Zimmer et al., 2020). And the deadlock occurs in the 2D mesh usually. When the requested number of packets is more than that of the packet buffer size, the cores wait each other infinitely, thus deadlock occurs. To avoid deadlock and optimize the communication latency and energy in physical mapping, reinforcement learning (Ma et al., 2019; Barrett et al., 2020; Feng et al., 2020; Cappart et al., 2021; Mazyavkina et al., 2021) is used by some researchers (Wu et al., 2020; Myung et al., 2021). Moreover, Figure 3 explains the communication latency in a multi-core architecture after physical mapping (Amin et al., 2020). Because the 8-th and the 9-th cores send data to the 11-th core concurrently, the 10th core is crossed twice by them due to the physical route. Thus, the latency is generated by the 10th core.

There are many heuristic solutions to physical mapping, such as the genetic algorithm (GA) (Lei and Kumar, 2003; Zhou et al., 2006) and the simulated annealing (SA) (Ma et al., 2020) algorithm. Some teams (Davies et al., 2018; Shao et al., 2019; Zimmer et al., 2020) also use the greedy algorithm to optimize the communication latency and energy. We use *t*_*add*_2_ to denote the additional computation latency of a core when considering the physical mapping. It can be obtained by


(7) $$\begin{array}{l} t_{add\text{\_}2}=g_{1}\left(S_{row\text{\_}add}\right)+g_{2}\left(S_{col\text{\_}add}\right)\\ \;+g_{3}\left(S_{p\text{\_}add}\right)+g_{4}\left(S_{re\text{\_}add}\right). \end{array}$$

where *g*_*i*_(·) represents the function for processing above additional data under the condition of physical mapping. Similarly, we have *g*_*i*_(0) = 0.

## 3. Mapping limit

### 3.1. Logical mapping limit

For logical mapping, we introduce a theoretical description. We denote the sets of weights (W), input activations (IA), and output activations (OA) of the *i*-th and *j*-th core as *W*_*i*_, *W*_*j*_, *IA*_*i*_, *IA*_*j*_, *OA*_*i*_, and *OA*_*j*_, and further denote the storage volume of W and IA in each core as *S*_*W*_ and *S*_*IA*_, respectively. Then, the logical mapping can be described as


(8) $$\begin{array}{l} \forall i,\;\cup_{i}{IA}_{i}=IA,\;\cup_{i}W_{i}=W, \end{array}$$

(9) $$\begin{array}{l} \forall i,\;{OA}_{i}={IA}_{i}*W_{i},OA=IA*W, \end{array}$$

(10) $$\begin{array}{l} \forall i\neq j,\;\cup_{i}{OA}_{i}=OA,\;{OA}_{i}\cap {OA}_{j}=\emptyset , \end{array}$$

(11) $$\begin{array}{l} S_{IA}+S_{W}\leq S_{mem}, \end{array}$$

where *S*_*mem*_ represents the total memory volume of a core. The non-overlap of OA indicates each output activation is calculated only once. Because OA will be transmitted to the IA memory of the cores for the next layer, *S*_*mem*_ does not take *S*_*OA*_ into account.

The additional storage overhead for a core generated in partition can be calculated by:


(12) $$\begin{array}{l} S_{col\text{\_}add}=\frac{H_{in}C_{in}}{Jm}\left(K_{w}-s\right)\cdot \mu \left(I-2\right), \end{array}$$

(13) $$\begin{array}{l} S_{row\text{\_}add}=\frac{W_{in}C_{in}}{Im}\left(K_{h}-s\right)\cdot \mu \left(J-2\right), \end{array}$$

(14) $$\begin{array}{l} S_{re\text{\_}add}=\frac{H_{out}W_{out}C_{out}}{IJm}\cdot \mu \left(n-2\right), \end{array}$$

(15) $$\begin{array}{l} S_{p\text{\_}add}=\frac{H_{out}W_{out}C_{out}}{IJm}\cdot \frac{b_{p}}{b}\cdot \mu \left(m-2\right). \end{array}$$

where μ(·) represents the unit step function, *s* represents the stride of the filter, *H*_*in*_, *W*_*in*_, *H*_*out*_, and *W*_*out*_ represent the height and width sizes of IA and OA, respectively. *k*_*w*_ and *k*_*h*_ are the width and height sizes of each weight kernel. And *b*_*p*_ and *b* represent the bit-width of *psum* and IA, respectively. The logical mapping limit means that the logical mapping does not produce any additional storage overhead, which can be described as


(16) $$\begin{array}{l} \forall i\neq j,IA_{i}\cap IA_{j}=\emptyset ,\;W_{i}\cap W_{j}=\emptyset \end{array}$$

(17) $$\begin{array}{l} S_{add}=0. \end{array}$$

When we approach the logical mapping limit, the values of *S*_*row*_*add*_, *S*_*col*_*add*_, *S*_*p*_*add*_, *S*_*re*_*add*_, *t*_*add*_1_, and *t*_*add*_2_ should be zero.

### 3.2. Physical mapping limit

After the logical mapping stage, the logical cores would be mapped onto the physical cores in a real chip. With the aforementioned graph representation, we optimize the average communication latency (*L*) and power consumption (*P*) without deadlock. The physical mapping limit here implies all logical cores are physically placed very close, especially being neighbors with Manhattan distance equal to one, which can be described as


(18) $$\begin{array}{l} \forall v_{i},v_{j},M_{ij}=1. \end{array}$$

Then, the communication cost can be reduced to


(19) $$\begin{array}{l} C_{cost}=\sum_{\forall v_{i},v_{j}\in V}c_{ij}\times M_{ij}=\sum_{\forall v_{i},v_{j}\in V}c_{ij}. \end{array}$$

Because W and IA must be put in cores, the minimum of *C*_*cost*_ is the sum of *W* and *IA*. Now, the communication cost can be given as follows:


(20) $$\begin{array}{l} C_{cost}=\sum_{\forall v_{i},v_{j}\in V}c_{ij}\\ \;=\sum_{\forall v_{i}\in V}\left(IA_{i}+W_{i}\right)=IA+W. \end{array}$$

The average communication latency and power consumption can be the communication latency and power consumption by transmitting a routing packet between two neighboring cores due to *M*_*ij*_ = 1.

In short, integrating the logical mapping limit and the physical mapping limit, the overall mapping limit follows


(21) $$\begin{array}{l} \forall i\neq j,IA_{i}\cap IA_{j}=\emptyset ,\;W_{i}\cap W_{j}=\emptyset , \end{array}$$

(22) $$\begin{array}{l} S_{add}=0, \end{array}$$

(23) $$\begin{array}{l} \forall v_{i},v_{j},M_{ij}=1. \end{array}$$

To approach the mapping limit, a closed-loop mapping strategy is proposed in the next section.

## 4. Approaches

### 4.1. Closed-loop mapping strategy

**Figure 4:**
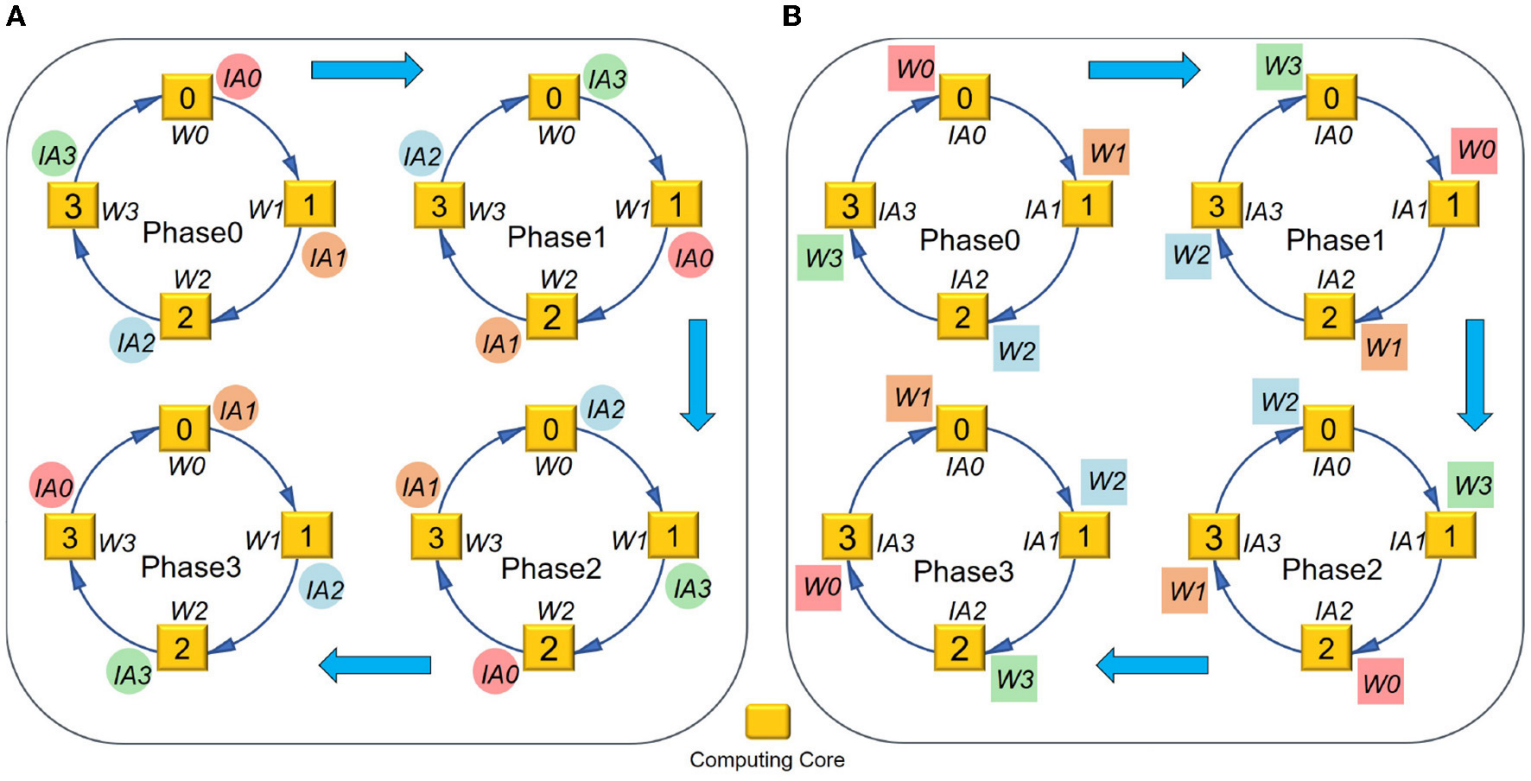
Closed-loop mapping based on **(A)** IA or **(B)** W.

To approach the logical mapping limit, we propose a closed-loop mapping strategy with two forms. As illustrated in Figure 4, one form is based on IA, and the other is based on W. Taking four cores and the IA-based form as an example (see Figure 4A), the computing process can be described as follows. In the first phase, each core performs the convolution operation between *IA*_*i*_ and *W*_*i*_. At the end of the first phase, each core keeps its *W*_*i*_ stationary and sends its *IA*_*i*_ to the downstream core. In the next phase, each core performs the convolution operation between *W*_*i*_ and its newly received IA. This loop would be closed when all cores have performed a complete convolution operation between its local *W*_*i*_ and all IAs. In this example, the loop needs four phases to close, and then we can get all OAs distributed in the four cores. The computing process can be summarized as


(24) $$\begin{array}{l} OA_{\left(i-t+N\right)\%N}=\overset{N-1}{\underset{t=0}{\sum }}IA_{\left(i-t+N\right)\%N}*W_{i}. \end{array}$$

where *N* denotes the number of cores used for the layer and *t* is the index of phases. It can be seen that the above mapping strategy does not consume any additional memory overhead, satisfying the logical mapping limit given in Equations (21)–(22). For the W-based closed-loop mapping, the overall flow is similar. The only difference is that each core keeps IA stationary and exchanges W between cores.

In order to implement the closed-loop mapping on hardware, a 4D partition with synchronous and asynchronous methods is proposed for logical mapping, which is more flexible and general than the existing 2D synchronous partition. Here “4D” refers to *C*_*in*_, *C*_*out*_, and two dimensions of each feature map.

**Figure 5:**
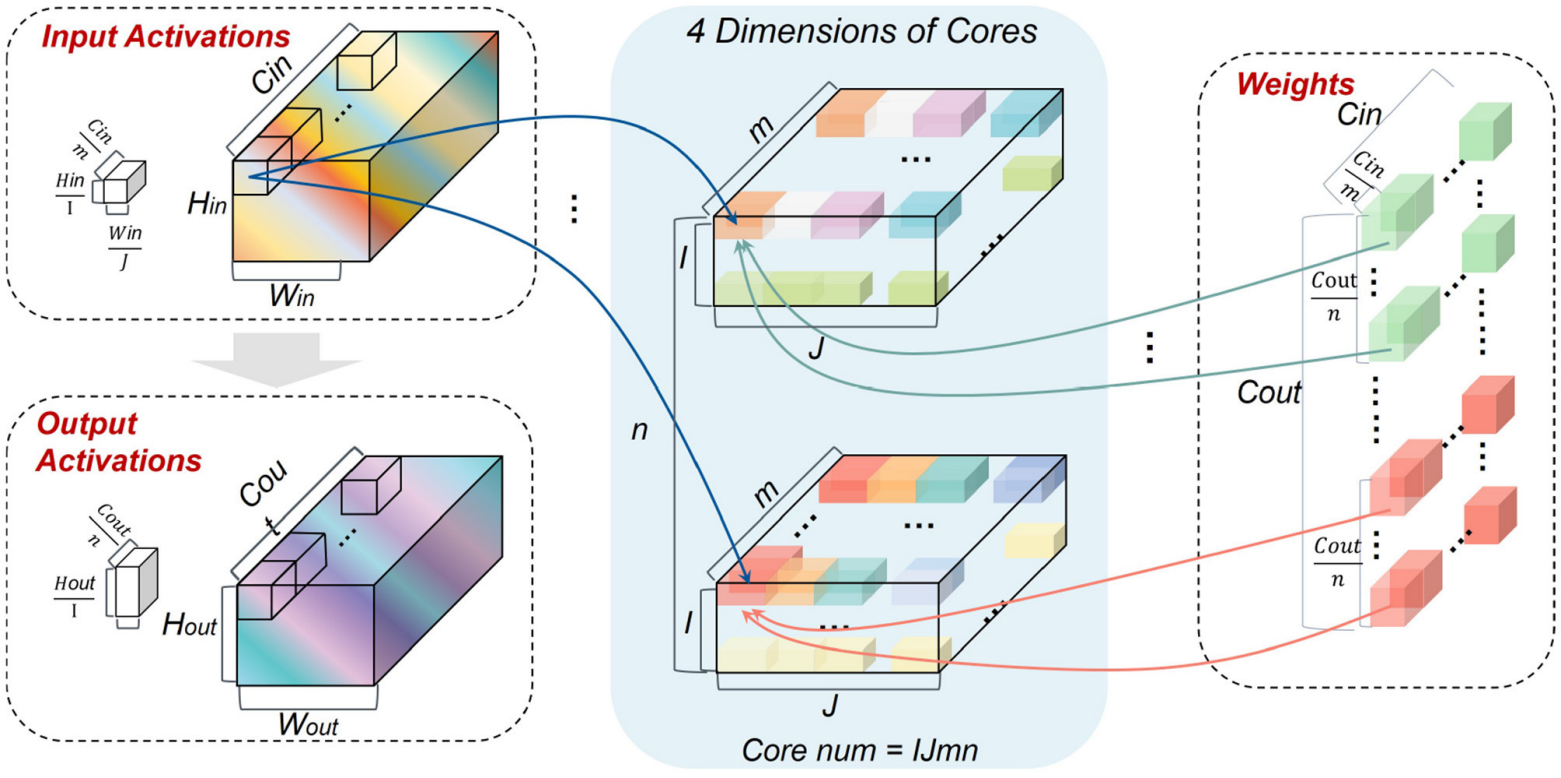
Illustration of the 4D synchronous mapping.

First, we try the 4D synchronous partition, as illustrated in Figure 5. Note that *I*, *J*, *m*, and *n* represent the number of partition groups in feature map height, feature map width, *C*_*in*_, and *C*_*out*_ dimensions, respectively. *k*_*w*_ and *k*_*h*_ are the width and height sizes of each weight kernel. In the synchronous partition, W should be broadcasted along the feature map dimensions *I*×*J* times, and IA should be broadcasted along the output channel dimension *n* times. Therefore, the redundancy of storage caused by this partition is


(25) $$\begin{array}{l} S_{sync}=\frac{n\left(H_{in}W_{in}C_{in}\right)+IJ\left(k_{w}k_{h}C_{in}C_{out}\right)}{H_{in}WinC_{in}+k_{h}k_{w}C_{in}C_{out}}-1. \end{array}$$

Moreover, the number of allocating cores is


(26) $$\begin{array}{l} N=IJmn. \end{array}$$

The resulting storage overheads for IA and W in each core should be


(27) $$\begin{array}{l} S_{IA}=\frac{H_{in}W_{in}C_{in}}{IJm},S_{W}=\frac{k_{w}k_{h}C_{in}C_{out}}{mn}. \end{array}$$

In short, the additional storage overhead on hardware given the 4D synchronous partition can be


(28) $$\begin{array}{l} S_{hw\text{\_}add}=\left(IJ-1\right)k_{w}k_{h}C_{in}C_{out}+\left(n-1\right)H_{in}W_{in}C_{in}+IJmnS_{add}. \end{array}$$

Apparently, Equation (21) can only be satisfied under the condition *IJn* = 1, but Equation (22) cannot be satisfied under this case because *psum*s exist. Therefore, the synchronous 4D partition fails to approach the logical mapping limit.

**Figure 6:**
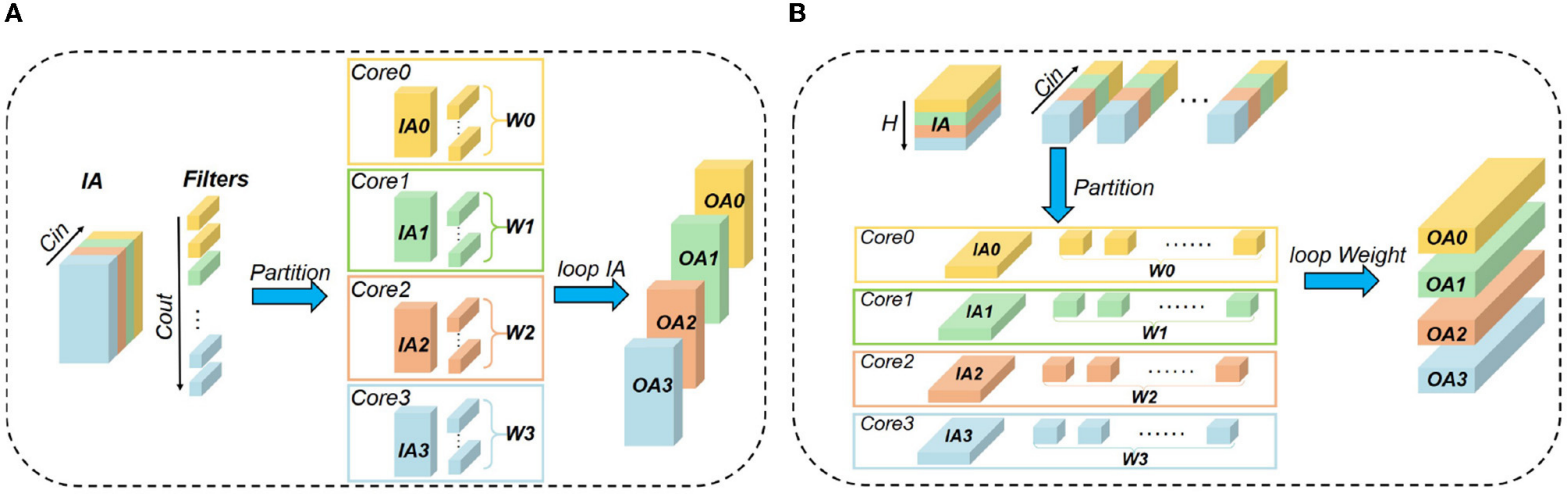
Illustration of the 4D asynchronous closed-loop mapping: **(A)** exchanging IA as Figure 4A; **(B)** exchanging W as Figure 4B.

In order to approach the logical mapping limit described in Equations (21)–(22), we further propose an asynchronous partition method based on the closed-loop mapping strategy. Corresponding to the IA-based closed-loop mapping, the asynchronous partition method selects *C*_*in*_ of IA and *C*_*out*_ of W to partition. Taking *N* = 4 as an example, it can be seen from Figure 6A that both *C*_*in*_ of IA and *C*_*out*_ of W are partitioned into *m* = *n* = *N* groups. Then, the resulting IA and W in each core can satisfy Equation (21) without duplication. The reshaping overhead does not exist because the shape of OA is consistent with that of IA. Because *psum*s can be accumulated locally, the *psum* communication also does not exist. Therefore, all additional storage overheads are zero and Equation (22) is satisfied. For the W-based closed-loop mapping in Figure 6B, the overall idea is similar to the IA-based case while *H*_*in*_ of IA and *C*_*in*_ of W are selected to partition. For the asynchronous closed-loop mapping, the storage overheads for IA and W in each core are


(29) $$\begin{array}{l} S_{IA}=\frac{H_{in}W_{in}C_{in}}{N},S_{W}=\frac{k_{w}k_{h}C_{in}C_{out}}{N}. \end{array}$$

With the above knowledge, we make an explanation for the words “synchronous” and “asynchronous.” In this work, “synchronous” means both the partitioning dimensions of IA and W involve *C*_*in*_. In contrast, “asynchronous” means the partitioning dimensions of IA and W are different, for example in Figure 6A partitioning IA along the *C*_*in*_ dimension while partitioning W along the *C*_*out*_ dimension, and in Figure 6B partitioning IA along the *H*_*in*_ dimension while partitioning W along the *C*_*in*_ dimension. In the asynchronous closed-loop mapping, one of IA and W in each core has a complete *C*_*in*_ dimension, and the other is gradually acquired by exchanging data between cores without any redundant data copy.

### 4.2. Hamilton loop algorithm for physical mapping

**Figure 7:**
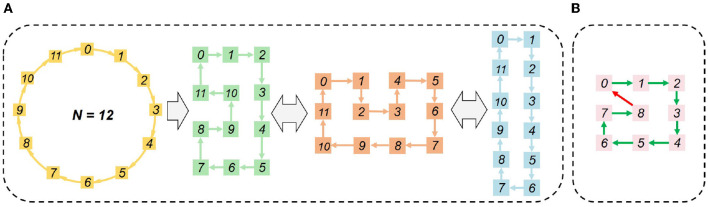
HLA-based physical mapping: **(A)** even number of cores; **(B)** odd number of cores.

To satisfy Equation (23) of the physical mapping limit, the Hamilton Loop Algorithm (HLA) is proposed for the closed-loop mapping strategy with asynchronous partition. Taking 12 cores as an example, it can be seen from Figure 7A that the Manhattan distance of every two logically neighboring cores equals 1, i.e., satisfying *M*_*ij*_ = 1 as given in Equation (23). The physical mapping form can be flexibly arranged according to the array form of the available physical cores, e.g., 4 × 3, 3 × 4, and 6 × 2. Notice that the number of cores cannot be odd, as illustrated in Figure 7B. In those cases, Equation (23) cannot be satisfied unless there is a diagonal communication path. Usually, only one Hamiltonian loop is needed. A fast algorithm is proposed to find a Hamiltonian loop, whose pseudo-codes are given in Algorithm 1.

**Algorithm 1:** Fast algorithm to find a Hamiltonian loop.

for i in range(m): //*row*
//*x direction communication distance*
dx[i][0] = 1 if i == 0 else 0
//*y direction communication distance*
dy[i][0] = 0 if i == 0 else -1
if n == 2:
dx[i][n - 1] = -1 if i == m-1 else 0
dy[i][n - 1] = -1 if i == m-1 else 0
else:
dx[i][n - 1] = 0 if i % 2 ==0 else -1
dy[i][n - 1] = 1 if i % 2 ==0 else 0
for j in range(1,n-1): //*column*
if j = =1 and i != m - 1:
dx[i][j] = 1 if i % 2 ==0 else 0
dy[i][j] = 0 if i % 2 ==0 else 1
else:
dx[i][j] = 1 if i % 2 ==0 else 1
dy[i][j] = 0

## 5. Experimental results

**Figure 8:**
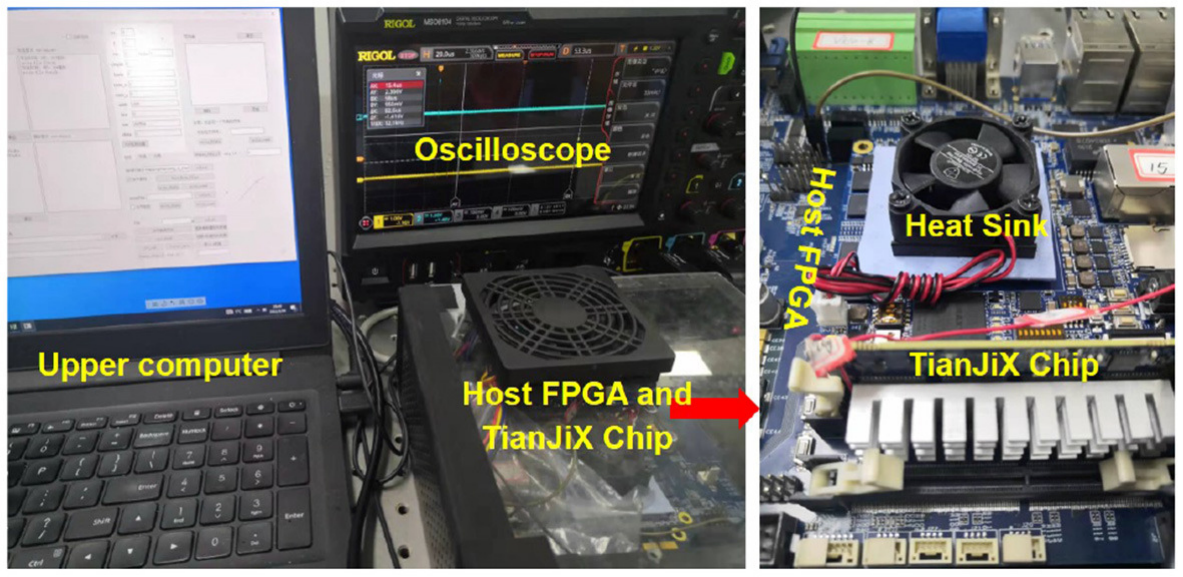
Testing system based on the TianjicX neuromorphic chip.

The mapping methods are implemented on a 28nm neuromorphic chip, TianjicX (Ma et al., 2022), which adopts a decentralized manycore architecture with 160 functional cores. Each core has 128 multipliers and accumulators (MACs) for parallel execution operations in neural networks. To maintain accuracy as high as possible, the precision for accumulating *psum*s is 32-bit. TianjicX supports the aforementioned 4D partition methods. The testing system includes a host computer, an Intel Arria 10 FPGA, four TianjicX chips, and an oscilloscope, as presented in Figure 8. The parameters and inputs of neural networks can be downloaded onto the chip by the configuration software on the host computer. The oscilloscope (RIGOL MSO8104) is used to measure the running time. Notice that the results of logical mapping are produced by the TianjicX simulator, while the results involving physical mapping are measured from the real chip.

### 5.1. Analysis of logical mapping

We focus our application measurements on the ResNet50 (He et al., 2016), which is often used to benchmark by many hardwares (Jiao et al., 2020; Zimmer et al., 2020; Jouppi et al., 2021). However, as we do not have an automatic mapping tool at the current stage, we select a portion of the ResNet50 convolutional network for experimental analyses. In essence, the methodology can be extended to the whole convolutional networks in principle. To optimize the running time of each dimension, the synchronous partition method is selected as a baseline for investigation. The benchmarking layers are the 5-th and 6-th layers of ResNet50. The dimension settings of synchronous mapping are listed in Table 1. *J*, *I*, *m* and *n* represent the numbers of partition groups in the width, height, input channel, and output channel dimensions, respectively. First, from Model 1 to Model 6, *I* × *J* is set to a constant, 28, to explore the influence of partitioning *I*, *J* on the running clocks. Second, from Model 7 to Model 12, *J* × *m* = *const* and *I* × *m* = *const* are set to compare the influence priority of *J*, *I*, and *m* in dimension partition. Third, from Model 13 to Model 15, the influence priority is further compared among *I*, *J*, *m*, and *n*. Finally, we analyze the impact of changing partition dimensions on the running latency and computing efficiency.

**Table 1:** Dimension settings of synchronous partition.

**No. of cores**	**J**	**I**	**m**	***n***	**Model**
28	28	1	1	1	Model 1
28	14	2	1	1	Model 2
28	7	4	1	1	Model 3
28	4	7	1	1	Model 4
28	2	14	1	1	Model 5
28	1	28	1	1	Model 6
56	28	1	1	2	Model 7
56	14	1	2	2	Model 8
56	7	1	4	2	Model 9
56	1	28	1	2	Model 10
56	1	14	2	2	Model 11
56	1	7	4	2	Model 12
56	28	1	2	1	Model 13
28	14	1	1	2	Model 14
28	1	28	1	1	Model 15

**Figure 9:**
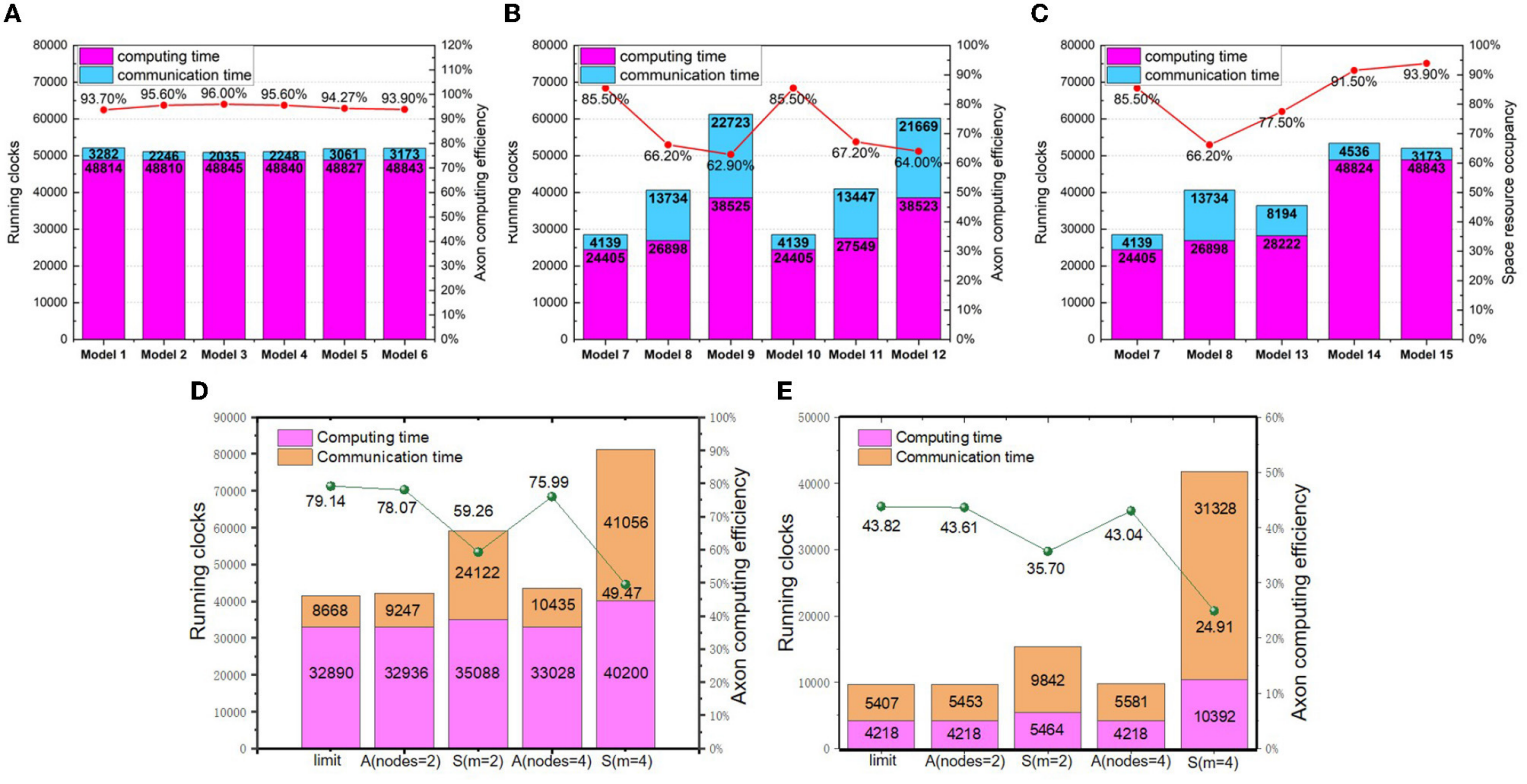
The logical mapping of running clocks and computing efficiency **(A)** synchronous partition of changing *I* and *J*; **(B)** synchronous partition of changing *m*, (*I*, *J*); **(C)** synchronous partition of changing *m*, *n*, and (*I*, *J*) **(D)** asynchronous partition with the 15-th layer of ResNet50; **(E)** asynchronous partition with the 16-th layer of ResNet50.

The experimental results of partitioning different dimensions are provided in Figure 9. From Figure 9A, it can be seen that the close the values between *J* and *I*, the shorter running time can be achieved. Meanwhile, we observe that the latency results of Model 1–6 present small variance, which implies that the partition of feature map dimensions has a negligible impact on the execution latency. From Figure 9B, it can be seen that the running time would be increased when we partition *C*_*in*_, which introduces additional accumulation of *psum*s and extra inter-core communication. As Figure 9C shows, although Model 7 introduces reshaping latency as *n* increases, it still reduces the total running clocks by 8000 owing to the decrease of *m*. It indicates that the partition of *C*_*in*_ has a larger impact on the running time than the partition of *C*_*out*_. Similarly, by comparing Model 14 and Model 15, we find the partition of *C*_*out*_ has larger impact than the partition of feature map dimensions. Overall, the accumulation and communication of *psum*s caused by partitioning *C*_*in*_ has the greatest impact on the execution latency, while reshaping caused by partitioning *C*_*out*_ has a greater impact than overlapping caused by partitioning feature map dimensions. With the above knowledge, it is possible to optimize execution latency by elaborating partition method.

As aforementioned, the asynchronous partition based on the closed-loop mapping strategy can approach the mapping limit. To demonstrate its superior performance, we compare the running latency and computing efficiency of both synchronous partition and asynchronous partition. We use two types of layers: one is the 15-th layer of ResNet50 with 3 × 3 weight kernels, and the other is the 16-th layer of ResNet50 with 1 × 1 weight kernels. The model settings for the two benchmarking layers are respectively listed in Tables 2, 3.

**Table 2:** Dimension setting of synchronous and asynchronous partition for the 15-th layer of ResNet50.

**No. of cores**	**J**	**I**	**m**	***n***	**Model**
28	7	1	1	4	limit
28	7	1	2	2	S(*m* = 2)
28	7	2	1	4	A(*#nodes* = 2)
28	7	1	4	1	S(*m* = 4)
28	7	4	1	4	A(*#nodes* = 4)

S, synchronous mapping; A, asynchronous mapping; No. of nodes, number of nodes in a closed loop.

**Table 3:** Dimension setting of synchronous and asynchronous partition for the 16-th layer of ResNet50.

**No. of cores**	**J**	**I**	**m**	***n***	**Model**
112	7	1	1	16	limit
112	7	1	2	8	S(*m* = 2)
112	7	2	1	16	A(*#nodes* = 2)
112	7	1	4	4	S(*m* = 4)
112	7	4	1	16	A(*#nodes* = 4)

S, synchronous mapping; A, asynchronous mapping; No. of nodes, number of nodes in a closed loop.

The experimental results are depicted in Figures 9D, E. Due to the limited number of primitive instructions in TianjicX, the maximum number of nodes in a closed loop cannot be larger than four. As Figure 9E presents, the running latency under asynchronous partition based on the closed-loop mapping is faster than that of synchronous mapping. For example, the running latency of the 16-th layer can be improved by 4.12 × under four nodes in a loop. Without the communication of *psum*s, the communication latency of asynchronous mapping can also be greatly reduced.

### 5.2. Analysis of HLA physical mapping

**Figure 10:**
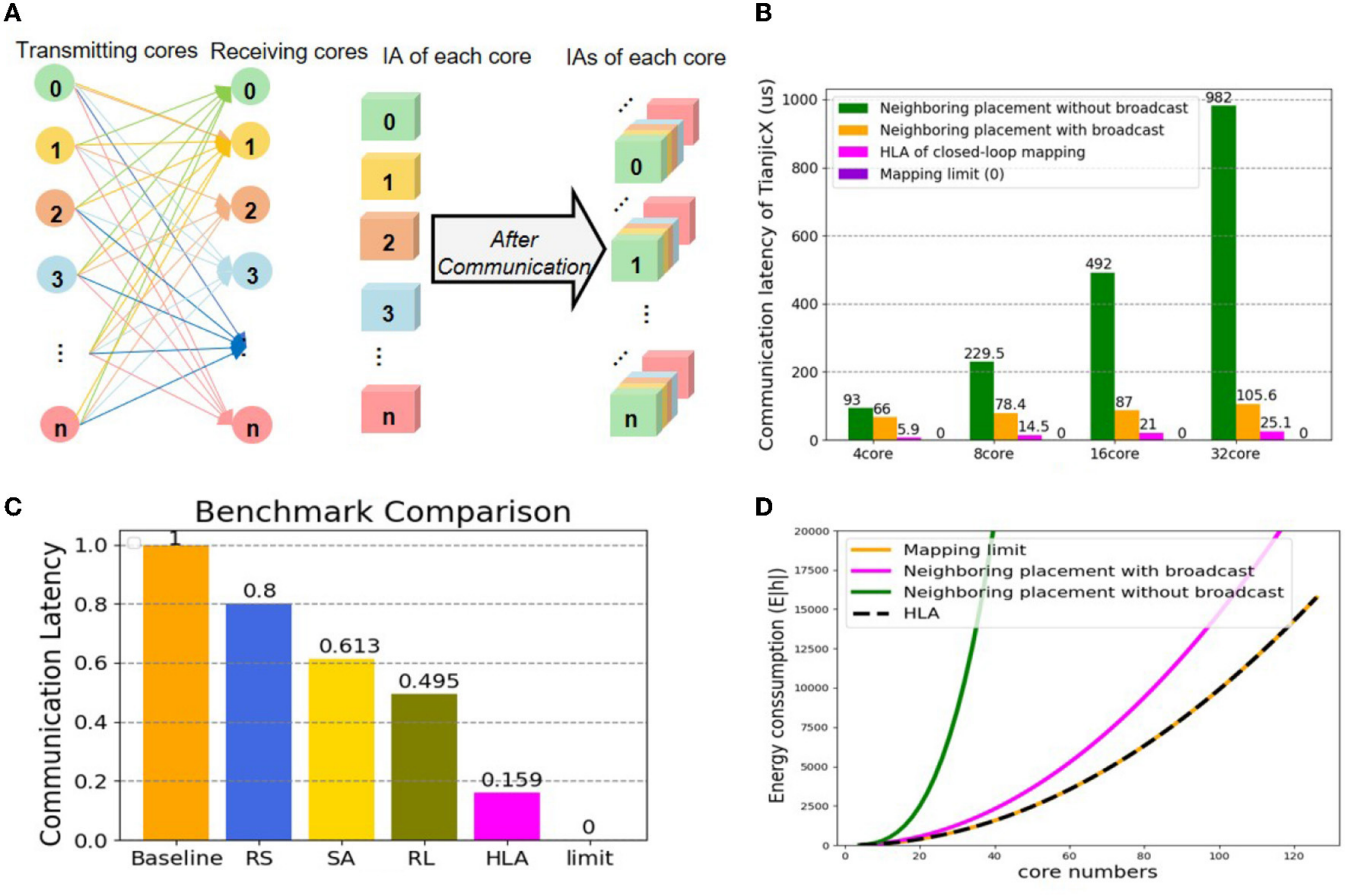
The physical mapping of latency and consumption **(A)** all-to-all communication between cores; **(B)** comparing communication latency between the neighboring placement, the mapping limit, and HLA; **(C)** comparing communication latency between prior methods, the mapping limit, and HLA; **(D)** comparing energy consumption between the neighboring placement, the mapping limit, and HLA.

To test the latency of HLA, we select all-to-all communication to conduct experiments. The 15-th layer of ResNet50 with 98KB parameters is the target workload. The all-to-all communication topology is illustrated in Figure 10A. The communicating latency is tested on TianjicX by enabling 4, 8, 16, or 32 cores. The energy consumption is estimated through simulation. Each case is tested with multiple physical mapping methods, including HLA, sequential neighboring placement with and without multicast (Myung et al., 2021), and several prior placement methods, including sequential placement (BS) (Wu et al., 2020), random search (RS) (Wu et al., 2020), simulated annealing (SA), and the RL-based approach (Wu et al., 2020). Due to the deadlock issue, we do not give the results of the zigzag physical mapping (Ma et al., 2020).

The communication latency results can be found in Figures 10B, C. As predicted, the communication latency of HLA is the shortest among all tested physical mapping methods, which is quite close to the mapping limit. The communication latency of HLA can be reduced by 4.22 × compared to the neighboring placement with broadcast, and reduced by 84.1, 80.1, 74.1, and 67.9% compared to BS, RS, SA, and RL, respectively. Due to the launching delay of chip primitives, the communication latency increases as the number of used cores grows. The communicating latency of HLA approaches that of the mapping limit if there is no launching overhead. When using the HLA physical mapping, all cores are parallel to communicate without deadlock.

Assuming the number of cores is *N* and the data of a core for communication is *V*, the total energy consumption of the mapping limit, HLA and the neighboring placement with broadcast and without broadcast can be calculated as follows:


(30) $$\begin{array}{l} E_{HLA}=E_{limit}=N\left(VE\text{|}h\text{|}\overset{N-1}{\underset{i=0}{\sum }}1\right)\\ \;=VE\text{|}h\text{|}\frac{N\left(N-1\right)}{T}, \end{array}$$

(31) $$\begin{array}{l} E_{N\text{\_}B}=VE\text{|}h\text{|}\overset{N-1}{\underset{i=0}{\sum }}i+N\left(VE\text{|}h\text{|}\overset{N-1}{\underset{i=0}{\sum }}1\right)\\ \;=VE\text{|}h\text{|}\frac{\left(N-1\right)\left(3N-2\right)}{2T}, \end{array}$$

(32) $$\begin{array}{l} E_{N\text{\_}W\text{\_}B}=VE\text{|}h\text{|}\overset{N}{\underset{i=0}{\sum }}\left(\overset{i}{\underset{t=0}{\sum }}t+\overset{N-i}{\underset{j=0}{\sum }}j\right)\\ \;=VE\text{|}h\text{|}\frac{2N^{3}-3N^{2}+N}{6T}, \end{array}$$

where *E*_*HLA*_, *E*_*limit*_, *E*_*N*_*B*_, and *E*_*N*_*W*_*B*_ represent the energy consumption of under HLA, the mapping limit, the neighboring placement with broadcast, and the neighboring placement without broadcast, respectively. The energy results can be found in Figure 10D. The energy consumption of all methods increases as the number of the allocated cores grows. Obviously, the energy consumption under the neighboring placement is much higher than that under the mapping limit, while the energy consumption under HLA is equal to that under the mapping limit.

In short, the HLA physical mapping based on the closed-loop mapping strategy shows significant superiority on reducing communication latency and energy consumption compared with other methods. More importantly, the HLA physical mapping can approach the mapping limit.

### 5.3. Integration of logical and physical mapping

**Figure 11:**
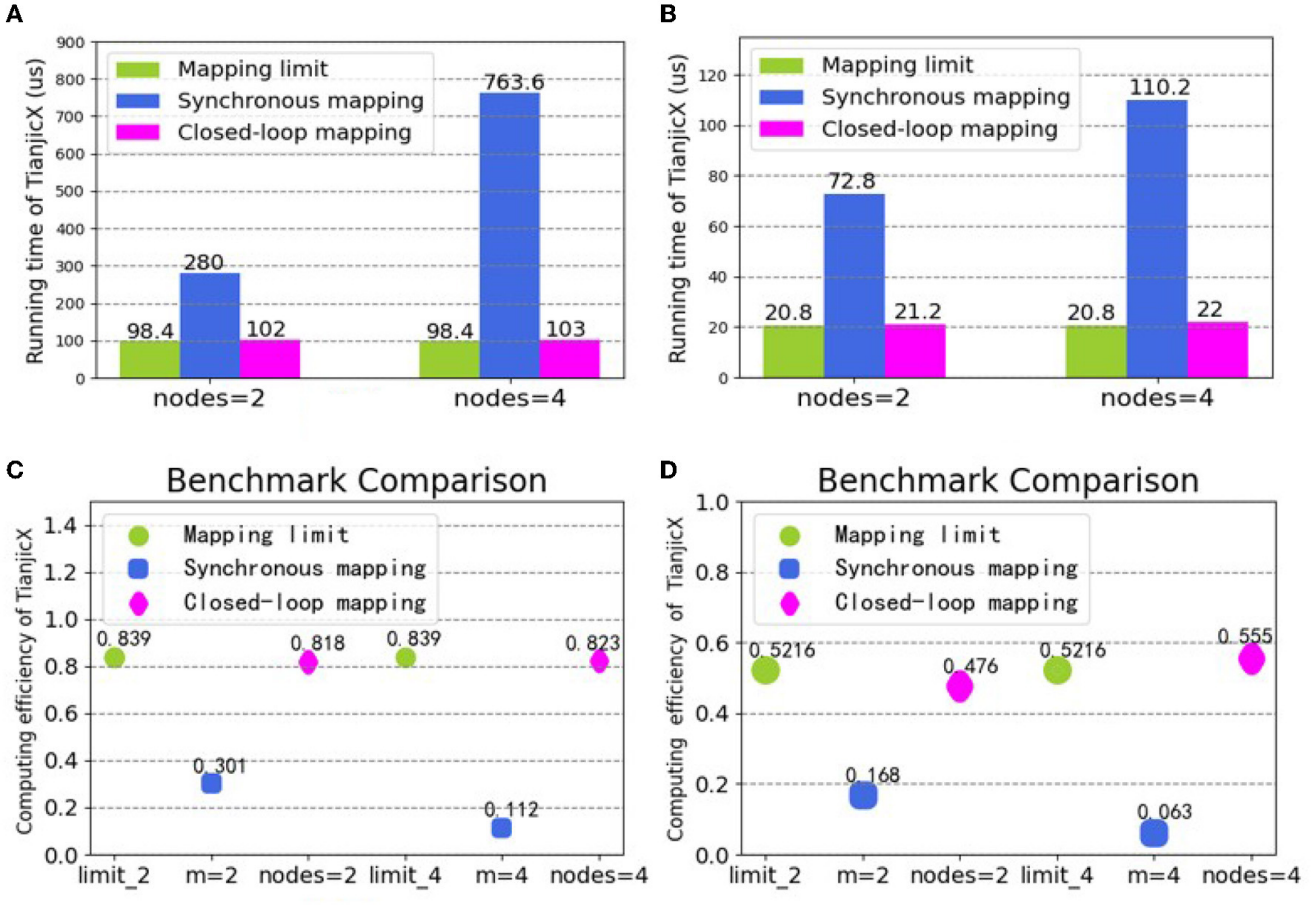
Running time and computing efficiency by integrating the logical and physical mapping under the closed-loop mapping strategy: **(A)** running time for the 15-th layer of ResNet50; **(B)** running time for the 16-th layer of ResNet50; **(C)** computing efficiency for the 15-th layer of ResNet50; **(D)** computing efficiency for the 16-th layer of ResNet50.

To demonstrate the performance of asynchronous logical mapping and HLA physical mapping based on the closed-loop mapping strategy, we deploy neural layers on TianjicX. The experimental results are provided in Figure 11. Again, Figures 11A, B evidence the superior latency of our closed-loop mapping strategy compared to the conventional synchronous mapping with *C*_*in*_ partition adopted by Simba (Shao et al., 2019; Zimmer et al., 2020), Tianjic (Pei et al., 2019; Deng et al., 2020), and other neural network accelerators (Han et al., 2016; Jouppi et al., 2017; Parashar et al., 2017; Shin et al., 2017; Chen et al., 2019). The better computing efficiency of the closed-loop mapping strategy is also evidenced by Figures 11C, D. Specifically, with four nodes in a closed loop, the running time can be reduced by 7.6 × for the 15-th layer of ResNet 50, and the computing efficiency can be improved by 8.8 × for the 16-th layer. The proposed closed-loop mapping strategy implemented by integrating the asynchronous partition and the HLA placement can approach the mapping limit.

## 6. Conclusion and discussion

In this work, we propose the mapping limit concept for neuromorphic hardware based on the decentralized manycore architecture, which points out the resource saving upper limit during model deployment. To approach the mapping limit, we further propose the closed-loop mapping strategy that includes the asynchronous 4D partition for logical mapping and the HLA placement for physical mapping. Our experiments demonstrate the superiority of the proposed mapping methods. For example, compared to conventional synchronous *C*_*in*_ partition, our mapping methods improve the running time and computing efficiency by 7.6× and 8.8×, respectively, which can approach the mapping limit.

Generally, the mapping schemes for multi-core system can be divided into two processes: the first is the logical mapping process and the second is the physical mapping process. Furthermore, the logical mapping can be divided into two sets of models, which are synchronization and asynchronization. Most of the previous researches adopt the synchronization model based on the 2D mapping system (Shao et al., 2019; Ma et al., 2020; Wu et al., 2020; Myung et al., 2021), which only partitions the in-channel and out-channel of the neural network. And these researches focus on the physical mapping based on the 2D mapping system, while the 4D mapping system is a general model that has wider applications. Based on our 4D mapping system, we propose the mapping limit concept for the multi-core system. In the 4D mapping system, both the synchronization model and asynchronization model are demonstrated through intensive experiments. To achieve the mapping limit, we adopt the asynchronization mode to integrate the logical process and the physical process by the closed-loop mapping strategy.

Since the GPU is not a distributed architecture, the optimized result may be slightly rather than significantly improved in terms of energy consumption and computational speed. With the emergence of the decentralized architecture, the multi-core system is expected to be widely adopted due to its high-parallelism and memory locality (Painkras et al., 2013; Akopyan et al., 2015; Han et al., 2016; Parashar et al., 2017; Shin et al., 2017; Davies et al., 2018; Chen et al., 2019; Pei et al., 2019; Shao et al., 2019; Deng et al., 2020; Zimmer et al., 2020). Therefore, we are convinced that our proposed methods will provide a systematic solution to map neural networks onto multi-core systems, and provide guidance for further development of auto-mapping tools. Moreover, with the proposed mapping limit and the closed-loop mapping strategy, it is possible to build a general and efficient mapping framework for multi-core system in the future.

## Data availability statement

The raw data supporting the conclusions of this article will be made available by the authors, without undue reservation.

## Author contributions

SW proposed the idea, designed and did the experiments, and wrote the manuscript. SW and QY conducted the algorithm modeling work, contributed to the analysis, and interpretation of results. SW, QY, and TX conducted the design and implementation of the hardware testing platform. CM led the discussion and revised it. JP directed the project and provided overall guidance. All authors contributed to the article and approved the submitted version.
